# Neuroimaging and Machine Learning in OCD: Advances in Diagnostic and Therapeutic Insights

**DOI:** 10.3390/brainsci15101106

**Published:** 2025-10-14

**Authors:** Norah A. Alturaiqi, Wijdan S. Aljebreen, Wedad Alawad, Shuaa S. Alharbi, Haifa F. Alhasson

**Affiliations:** Department of Information Technology, College of Computer, Qassim University, Buraydah 52571, Saudi Arabia; 451214501@qu.edu.sa (N.A.A.); 451214502@qu.edu.sa (W.S.A.); wmaoad@qu.edu.sa (W.A.); shuaa.s.alharbi@qu.edu.sa (S.S.A.)

**Keywords:** artificial intelligence, diagnostic imaging, diagnosis, deep learning, deep neural networks, machine learning, medical image processing

## Abstract

Background/Objectives: Obsessive–Compulsive Disorder (OCD) is a chronic mental health condition characterized by intrusive thoughts and repetitive behaviors. Traditional diagnostic methods rely on subjective clinical assessments, delaying effective intervention. This review examines how advanced neuroimaging techniques, such as Magnetic Resonance Imaging (MRI) and Diffusion Tensor Imaging (DTI), integrated with machine learning (ML), can improve OCD diagnostics by identifying structural and functional brain abnormalities, particularly in the cortico-striato-thalamo-cortical (CSTC) circuit. Methods: Findings from studies using MRI and DTI to identify OCD-related neurobiological markers are synthesized. Machine learning algorithms like Convolutional Neural Networks (CNNs) and Support Vector Machines (SVMs) are evaluated for their ability to analyze neuroimaging data. The role of transfer learning in overcoming dataset limitations and heterogeneity is also explored. Results: ML algorithms have achieved diagnostic accuracies exceeding 80%, revealing subtle neurobiological markers linked to OCD. Abnormalities in the CSTC circuit are consistently identified. Transfer learning shows promise in enhancing predictive modeling and enabling personalized treatment strategies, especially in resource-constrained settings. Conclusions: The integration of neuroimaging and ML represents a transformative approach to OCD diagnostics, offering improved accuracy and biologically informed insights. Future research should focus on optimizing multimodal imaging techniques, increasing data generalizability, and addressing interpretability challenges to enhance clinical applicability. These innovations have the potential to advance precision diagnostics and support more targeted therapeutic interventions, ultimately improving outcomes for individuals with OCD.

## 1. Introduction

Obsessive–Compulsive Disorder (OCD) is a chronic and debilitating psychiatric condition characterized by recurrent, intrusive, and unwanted thoughts, images, or impulses (obsessions), often accompanied by repetitive behaviors or mental acts (compulsions). These compulsions are performed to alleviate distress or prevent perceived harmful outcomes, despite the individual recognizing them as excessive or irrational [1]. In Saudi Arabia (SA), OCD affects approximately 1.3 million adults, accounting for 4% of the population [2]. Common compulsions include excessive washing, cleaning, checking, or organizing and arranging. A notable subtype of OCD, known as scrupulosity, involves a pathological focus on religious or moral correctness and is recognized in diagnostic literature [3].

OCD is a highly heterogeneous condition, with symptoms varying widely in presentation and severity. Some individuals primarily experience intrusive obsessive thoughts, while others exhibit compulsive behaviors that can significantly impair daily functioning. The severity of symptoms ranges from mild, with little impact on activities of daily living (ADL), to severe, resulting in profound disability. In some cases, OCD symptoms may overlap with restricted and repetitive behaviors and interests (RRBIs) observed in other neuropsychiatric disorders, further complicating diagnosis. The course of OCD is typically chronic, with symptom severity fluctuating over time. This variability reflects the complexity of the disorder, reinforcing the need for accurate diagnosis and individualized treatment strategies.

Recent advancements in neuroimaging techniques, particularly Magnetic Resonance Imaging (MRI), have provided valuable insights into the brain structure and connectivity associated with OCD. MRI, especially Diffusion Tensor Imaging (DTI), allows for the examination of white matter integrity and neural pathways, which have been shown to be altered in OCD. These imaging techniques provides a more precise understanding of the brain’s role in OCD, aiding in the identification of neurobiological markers.

Machine learning (ML) techniques are increasingly being integrated into the diagnostic process of OCD. Using MRI scan data sets, ML algorithms can automate the identification of patterns in brain structure that differentiate individuals with OCD from healthy controls. Among these techniques, support vector machines (SVMs) have consistently achieved diagnostic accuracies greater than 80% in previous studies of neuroimaging data [4]. This robustness suggests the potential of these techniques for future clinical application, pending further validation in real-world settings. These techniques can enhance diagnostic accuracy by identifying subtle neural alterations that may not be visible through traditional methods. Additionally, ML can support the development of predictive models, improving early detection and personalized treatment strategies.

Despite advances in understanding OCD and the potential for MRI and ML to revolutionize diagnosis, significant gaps remain in developing standardized diagnostic criteria and effective clinical applications. This paper aims to explore the potential of MRI and ML in the diagnosis of OCD, focusing on how these technologies can enhance diagnostic precision and inform personalized treatment strategies. However, significant gaps remain in understanding how these technologies can be effectively integrated. Although prior reviews (e.g., Li et al. [5], Masjoodi et al. [6]) have examined the role of neuroimaging and ML in OCD, these studies have largely focused on individual imaging modalities and traditional classification algorithms. This fragmented approach leaves critical gaps in understanding how multimodal imaging and advanced ML techniques can be integrated to improve diagnostic accuracy and treatment outcomes. Our review addresses these gaps by synthesizing findings across multiple imaging modalities, emphasizing the utility of emerging ML techniques such as Vision Transformers and Graph Neural Networks, and proposing a framework for clinical translation. By doing so, we aim to advance the field toward more personalized and effective diagnostic and therapeutic strategies for OCD.

In this review, a narrative perspective is taken in order to integrate recent progress in the context of neuroimaging and ML applications to OCD. Here, we summarize both DTI, which is most relevant because white matter abnormalities can be detected, and structural and Functional Magnetic Resonance Imaging (fMRI) to create a comprehensive general picture on the role of these imaging modalities in diagnostic and therapeutic progress.

## 2. Background and Fundamentals of OCD

OCD is a mental health condition characterized by persistent, intrusive thoughts (obsessions) and repetitive behaviors or mental acts (compulsions) that individuals feel compelled to perform [7,8,9]. These obsessions typically induce significant anxiety and distress, while the compulsions are employed as coping mechanisms to mitigate discomfort. However, these compulsions can become excessive and disruptive, impairing various aspects of an individual’s life, including social relationships, academic performance, employment, and family dynamics [10,11].

Despite its high prevalence, OCD continues to pose significant challenges in both diagnosis and treatment. Traditional diagnostic methods rely heavily on subjective clinical assessments and self-reported symptoms, which can vary widely across cultures and individuals [5,12]. Research indicates that OCD arises from a complex interplay of genetic, neurobiological, environmental, and psychological factors. Globally, the prevalence of OCD is estimated to range from 2% to 3%, with minimal variation between countries and regions [5]. Genetic studies suggest that individuals with a family history of OCD are at an elevated risk, highlighting a hereditary component. Furthermore, dysregulations in neuronal circuits involving serotonin and other neurotransmitters have been implicated, alongside structural and functional abnormalities in key brain regions such as the orbitofrontal cortex, basal ganglia, and anterior cingulate cortex [12,13].

Research has identified variations in brain structure related to OCD, particularly within the cortico-striato-thalamo-cortical (CSTC) circuit. There are a number of studies that highlighted reduced GM volume within the CSTC circuit and increased in subcortical regions like striatum [14,15,16]. These contradictory results could be due to differences in methodology, such as differences in acquisition parameters of MRI, statistical analysis technique, and the type of sample used between these studies. For instance, Boedhoe et al. [15] noted structural variability across neuroimaging studies of OCD and the influence of small sample sizes and analytic heterogeneity. Esteban et al. [17] stressed the impact of preprocessing pipelines on morphometric measurements in clinical samples. In order to address these discrepancies, it is important to inherit standardized imaging protocols and to facilitate collection of more diverseand larger datasets to enhance reliability and generalization capabilities of studies in OCD [18,19]. There have also been development of tools, such as BigBrainWarp, aiming at enhancing cross-study harmonization [20].

Advancements in neuroimaging techniques, particularly MRI and Positron Emission Tomography (PET), have significantly enhanced our understanding of OCD by identifying changes in brain structure and function associated with the disorder [21]. These imaging methods have provided insights into how dysfunctions in decision-making, emotional regulation, and threat perception pathways may contribute to OCD.

Additionally, environmental factors, such as chronic stress, traumatic experiences, or infections like streptococcal infections during childhood, have been implicated in the onset of OCD. Psychological theories further propose that personality traits, including perfectionism and heightened responsibility, may predispose individuals to the disorder [22].

This multifaceted understanding emphasizes that OCD is likely the result of an interaction between biological vulnerabilities and environmental triggers rather than being attributable to a single causative factor. Ongoing research into genetic, neurobiological, and environmental influences, along with innovations in brain imaging techniques, holds promise for enhancing diagnostic accuracy and developing more effective treatment strategies for OCD.

### 2.1. Advances in Brain Imaging Techniques for OCD

Imaging of the brain is crucial to making accurate diagnosis in patients presenting with psychiatric complaints. In psychiatry, the WT is not just helpful for diagnosing mental illnesses but also for generating new treatment strategies. Imaging tests of the brain are required to differentiate diseases including depression from brain tumors or neurodegenerative diseases and to discover anatomic changes related to OCD [23]. Delays in diagnosis can lead to a lack of treatment of the symptoms that can cause the condition to become more severe with increasing time [24,25]. Thus, effective OCD management is contingent upon timely and accurate identification, and intervention.

However, while ML approaches applied to brain imaging data offer promising diagnostic tools, classification accuracies for OCD vary widely—ranging from 70% to 95% across different datasets [26,27]. These difficulties emphasize the pressing requirement for standardization of methodologies and external validation to ensure that diagnostic models reflect different populations [17,28].

Various brain imaging modalities, such as computed tomography (CT), X-rays, and magnetic resonance imaging (MRI), are utilized in clinical practice. Among these, MRI stands out due to its advanced capabilities and its pivotal role in the diagnosis and research of Obsessive–Compulsive Disorder (OCD) [16]. MRI techniques have consistently demonstrated high efficacy in studying and diagnosing OCD by offering detailed insights into structural and functional brain abnormalities associated with the disorder. The most commonly employed and effective MRI techniques for OCD diagnosis include the following.

#### 2.1.1. Structural Magnetic Resonance Imaging

fMRI is a vital tool for examining anatomical changes in the brain, such as alterations in gray matter volume and cortical thickness. In OCD, structural MRI studies have consistently identified reductions in gray matter volume in critical brain regions, including the orbitofrontal cortex, anterior cingulate cortex, and basal ganglia. For example, Menzies et al. [29] reported significant gray matter reductions in the orbitofrontal cortex, while Garibotto et al. [30] highlighted structural changes in the anterior cingulate cortex and basal ganglia, supporting the involvement of these regions in OCD pathophysiology. Such findings underscore the structural abnormalities that may underlie key OCD symptoms, including compulsivity and intrusive thoughts. From a clinical perspective, structural MRI is essential for detecting these abnormalities, thereby facilitating diagnosis and enhancing our understanding of the neural mechanisms contributing to OCD.

Voxel-based morphometry (VBM) is an advanced neuroimaging analysis technique used to detect regional differences in brain anatomy by quantifying gray matter volume and density on a voxel-wise basis. VBM analyses in OCD have revealed structural alterations such as decreased gray matter volume in the orbitofrontal cortex and increased volume in subcortical structures like the striatum [13]. These findings provide further support for the involvement of cortico-striatal circuits in the pathophysiology of OCD and complement the results obtained from conventional structural MRI studies.

#### 2.1.2. Diffusion Tensor Imaging

DTI is a specialized MRI technique that evaluates the integrity and connectivity of white matter tracts within the brain. In OCD, DTI studies have consistently revealed disruptions in white matter pathways, particularly within the CSTC circuit. These disruptions suggest impaired communication between brain regions that are crucial for regulating compulsive behaviors and intrusive thoughts [30]. Clinically, DTI provides a unique advantage by detecting microstructural abnormalities in white matter that may not be visible with conventional MRI. This capability offers valuable insights into the neural underpinnings of OCD and the associated disruptions in brain connectivity.

#### 2.1.3. Functional Magnetic Resonance Imaging

fMRI is a well-established imaging modality that captures dynamic brain activity by measuring changes in blood oxygenation levels (BOLD signal). In the OCD literature, abnormal activation patterns have been detected using fMRI in regions such as the orbitofrontal cortex, anterior cingulate cortex, and basal ganglia—key components of the CSTC circuit [21]. These dysfunctions are thought to underlie cognitive processes related to decision-making, emotional regulation, and threat perception in individuals with OCD. In particular, task-based fMRI paradigms have been used to scan the brain while patients perform specific tasks designed to elicit obsessive thoughts or compulsive behaviors. In OCD, task-based fMRI studies have demonstrated overactivation in regions such as the orbitofrontal cortex and anterior cingulate cortex during OCD-specific tasks. This hyperactivity highlights the neural correlates of symptom expression and reflects the heightened activity within the CSTC circuit [31]. Clinically, task-based fMRI is valuable for understanding how OCD symptoms are associated with specific brain activity patterns. It provides insights into the functional mechanisms that underlie the disorder, helping to inform potential therapeutic strategies.

#### 2.1.4. Magnetic Resonance Spectroscopy

Magnetic Resonance Spectroscopy (MRS) is a neuroimaging technique that quantifies the concentration of specific brain metabolites, such as glutamate, gamma-aminobutyric acid (GABA), N-acetylaspartate, myoinositol, and choline, which are closely associated with neuronal function and neurotransmitter activity. In the context of OCD, MRS studies have consistently reported elevated glutamate levels in regions such as the striatum and anterior cingulate cortex, suggesting an imbalance between excitatory and inhibitory neurotransmission. Hazari et al. [31] demonstrated increased glutamate concentrations in the anterior cingulate cortex, while Maleki et al. [32] reported reduced GABA levels in cortical regions, further highlighting the role of neurotransmitter dysregulation in the pathophysiology of OCD. Recent studies have underscored the potential of Magnetic Resonance Spectroscopy (MRS) in identifying biochemical biomarkers for OCD. For instance, deviations in glutamate concentrations have been linked to compulsive behaviors, whereas reductions in GABA levels are associated with fear and intrusive, distressing thoughts [16]. These findings suggest that pharmacological interventions targeting glutamate and GABA pathways may hold promise for more effective treatment strategies.

From a clinical perspective, MRS provides valuable biochemical insights that complement findings from structural and functional imaging techniques. When integrated with fMRI, for instance, MRS offers a more comprehensive understanding of the relationship between neurotransmitter imbalances and abnormal brain activity patterns in the CSTC circuit. This integration could improve diagnostic precision and inform personalized treatment strategies, such as glutamate-modulating drugs or GABA-enhancing therapies [33].

MRS has also demonstrated utility in monitoring treatment response in OCD patients. For instance, studies have shown that pharmacological treatments, such as selective serotonin reuptake inhibitors (SSRIs) or glutamate modulators, can normalize glutamate levels in the anterior cingulate cortex, correlating with symptom improvement [32]. This positions MRS as a valuable tool for evaluating the biochemical effects of interventions and optimizing treatment plans.

Despite its promise, the clinical adoption of MRS faces challenges, including limited availability, high costs, and the need for standardization in acquisition protocols and metabolite quantification methods. Future research should prioritize harmonizing MRS methodologies across studies to enhance reproducibility and clinical applicability. Moreover, integrating MRS with ML approaches holds significant potential for identifying biochemical markers that predict treatment outcomes and facilitate early diagnosis.

#### 2.1.5. Multimodal Imaging

Multimodal imaging, which integrates techniques such as structural MRI, fMRI, and DTI, offers a comprehensive approach to understanding both structural and functional abnormalities in OCD. For instance, Yang et al. [34] emphasized the utility of combining structural and functional imaging techniques to analyze CSTC circuit disruptions, while Piras et al. [16] demonstrated the value of integrating DTI and fMRI for identifying white matter abnormalities and their functional implications in OCD. Studies employing multimodal imaging have significantly advanced our understanding of how white matter connectivity, gray matter structure, and functional brain activity interact in the disorder. These techniques have provided crucial insights into the neural circuits involved in OCD, particularly those associated with the CSTC circuit [34]. MRI techniques like DTI and fMRI are particularly effective for understanding OCD, as they reveal abnormalities in both structural connectivity (white matter) and functional brain networks. Meanwhile, MRS provides complementary biochemical data, and task-based fMRI identifies symptom-specific patterns of brain activity. Combining these techniques can lead to more accurate diagnosis, a better understanding of the disorder’s neural mechanisms, and improved treatment strategies. Clinically, multimodal imaging facilitates a holistic understanding of OCD, improving diagnostic accuracy, guiding treatment strategies, and advancing research into its complex neurobiology. The therapeutic implications of each imaging technique are summarized in Table 1 for clarity and ease of reference.

### 2.2. Applications of ML in Brain Imaging

The application of ML in the medical field has revolutionized the early and accurate diagnosis of a wide range of conditions [35]. By utilizing data-driven decision-making, ML enhances healthcare through early diagnosis, personalized treatment, and continuous monitoring of recovery [36]. ML algorithms excel at detecting complex patterns within large, multifaceted datasets, which makes them indispensable for analyzing medical images, electronic health records (EHRs), genomic data, and outputs from wearable devices. This capability has led to significant advancements in various medical domains, including ophthalmology, radiology, cardiology, and mental health [37].

One of the most impactful uses of ML in healthcare is in the analysis of medical images, including X-rays, MRIs, and CT scans, to detect abnormalities. For instance, convolutional neural networks (CNNs) have demonstrated exceptional accuracy in detecting conditions like brain tumors and skin cancer [38]. In the context of brain imaging, CNNs are particularly effective at identifying structural and functional abnormalities, which are critical for diagnosing conditions such as OCD, depression, and schizophrenia [39].

In addition to image analysis, ML is also pivotal in predictive analytics, where models analyze patient data and medical histories to predict hospital readmission rates or disease progression. This predictive power allows healthcare providers to offer proactive care, leading to improved patient outcomes [40]. Furthermore, natural language processing (NLP), a subset of ML, has enabled the extraction of clinical insights from unstructured text in medical records, enhancing treatment planning and diagnostic precision [41].

In mental health, ML is transforming diagnosis and prognosis by identifying biomarkers in neuroimaging data. Combining neuroimaging techniques with ML algorithms, researchers can identify brain regions and neural patterns associated with conditions like OCD, allowing for precise diagnoses and personalized treatment plans. Recent advancements in multimodal data fusion, which integrate structural MRI, fMRI, and DTI, have significantly improved classification accuracy by leveraging complementary information about brain abnormalities [42]. Additionally, self-supervised learning approaches, such as contrastive learning, have shown promise in extracting meaningful features from large-scale unlabeled neuroimaging datasets, addressing the challenges posed by limited labeled data [43]. Similarly, transfer learning techniques, like those utilizing Vision Transformers (ViTs) or Inception V3 models, have demonstrated their ability to improve diagnostic accuracy in OCD cases with small datasets [44,45].

Moreover, ML plays a key role in the analysis of behavioral data, including speech patterns and social media activity, offering non-invasive methods for detecting early signs of mental health issues such as anxiety and depression [46]. By enabling early identification, ML has the potential to slow the progression of these conditions, reduce treatment costs, and significantly improve patients’ quality of life.

## 3. Emerging Technologies in Brain Imaging and OCD Diagnostics

### 3.1. Transformer Architectures in Brain Network Analysis

Recent advancements in ML have introduced Transformer architectures as powerful tools in analyzing brain imaging data. Initially developed for natural language processing, Transformers have been adapted for vision-based tasks, such as MRI and fMRI analysis, through models like Vision Transformers (ViTs). These architectures excel at capturing global relationships in data, making them particularly useful for studying the connectivity between brain regions in OCD. For instance, Vision Transformers (ViTs) have shown superior performance in functional connectivity studies, identifying subtle abnormalities in the CSTC circuit associated with OCD [43,45]. Additionally, their attention mechanisms provide interpretability, helping researchers identify the brain regions most strongly linked to the disorder.

Chen [43] demonstrated the superiority of transformers, such as Vision Transformers (ViTs), in identifying functional connectivity abnormalities in OCD. By leveraging attention mechanisms, these models capture long-range dependencies across brain regions, outperforming traditional convolutional methods in accuracy and interpretability.

For example, Transformers have been applied to functional connectivity data to model long-range dependencies across brain regions, which are crucial for characterizing the CSTC circuit implicated in OCD. Unlike convolutional models, Transformers can dynamically focus on critical brain regions and connectivity patterns, enabling better diagnostic precision. Recent studies (e.g., refs. [43,45,47]) have demonstrated that Transformers outperform traditional ML approaches in tasks such as classifying OCD patients based on resting-state fMRI data. Furthermore, attention mechanisms within Transformers provide interpretability, allowing researchers to identify which regions contribute most to diagnostic predictions.

### 3.2. Graph Neural Networks (GNNs) for Brain Network Analysis

Another emerging technology is Graph Neural Networks (GNNs), which are uniquely suited for analyzing the non-Euclidean structure of brain networks. In this context, brain regions are represented as nodes, and their functional or structural connections form edges. This graph-based representation aligns well with the complex interactions within neural circuits, such as the CSTC loop. Recent studies have demonstrated that GNNs can model hierarchical relationships in brain connectivity data and detect subtle alterations in white matter and functional connectivity associated with OCD [42,48]. For example, BrainGNN, an interpretable GNN model, has been applied to fMRI data to classify OCD patients, achieving high accuracy and interpretability [48].

GNNs can model hierarchical relationships in brain connectivity data and have been successfully applied to detect subtle alterations in white matter and functional connectivity associated with OCD. For instance, GNNs have been used to classify OCD patients by extracting features from DTI and resting-state fMRI data, achieving high accuracy and interpretability [42,48,49]. Moreover, GNNs enable multi-scale analysis, providing insights into both local and global connectivity changes in OCD. This property is particularly valuable for identifying biomarkers and understanding the heterogeneity of OCD symptoms.

Many studies in OCD research are limited by small sample sizes, hindering the generalization of findings. Additionally, the lack of standardized imaging protocols and preprocessing steps complicates comparisons across studies. For example, variations in DTI preprocessing pipelines can lead to discrepancies in FA and MD measurements. While single-modality imaging (e.g., structural MRI) has been extensively studied, the integration of multimodal strategies, such as combining sMRI with functional imaging or spectroscopy, remains underexplored. Addressing these issues will require larger, multi-center datasets and standardized imaging protocols. Table 2 summarizes the consensus findings, conflicting results, and methodological weaknesses in OCD research, highlighting areas requiring further investigation.

## 4. Neuroimaging and ML Approaches for OCD Detection

This section provides an overview of key studies and methodologies relevant to OCD detection using MRI, with a particular focus on Diffusion Tensor Imaging (DTI) and ML techniques. It explores how integrating ML with advanced neuroimaging modalities has significantly enhanced the accuracy and timeliness of OCD diagnoses. Covering research conducted between 2015 and 2024, this section examines the evolution of approaches in detecting OCD, from traditional imaging methods to cutting-edge ML strategies. In the following subsections, we will delve into the classification of OCD using brain imaging data, the role of MRI and DTI in detecting OCD, and the diverse ML techniques employed. We will also explore the potential of traditional and transfer learning methods to assist in the diagnosis of OCD and support the treatment process for affected individuals.

### 4.1. Literature Search Strategy

To identify relevant studies for this review, we used the search strategy that is also aimed at identifying other studies on 10 March 2025 in the following databases: PubMed, Scopus, and Google Scholar. Full text Search was performed as following the combination of the terms of keyword and Boolean operators:

(“Obsessive-Compulsive Disorder” OR “OCD”) AND (“Diffusion Tensor Imaging” OR “DTI” OR “fMRI” OR “fMRI” OR “structural MRI” OR “sMRI”) AND (“machine learning” OR “deep learning” OR “artificial intelligence”).

The search was limited to articles published between January 2015 and March 2025 to capture recent developments. Only peer-reviewed journal articles written in English were considered. We excluded conference abstracts, editorials, case reports, and studies without sufficient methodological detail.

Titles and abstracts were reviewed for eligibility following removal of duplicates. Studies that were eligible for inclusion have been subjected to full-text review:

- Focus on the use of ML in neuroimaging for OCD diagnosis or treatment;
- Use of structural MRI, fMRI, DTI, or multimodal imaging;
- Availability of methodological details and performance metrics.

In total, 20 studies were included in the final review. A summary of the selected studies is presented in Table 3 and Table 4. More details about inclusion/Exclusion Criteria in Table 5.

### 4.2. Classification and Detection of OCD Using Neuroimaging Data

While this section focused on classifying OCD patients and detecting brain abnormalities, the following section explores the specific ML techniques that enable these advancements. Structural, functional, and connectivity alterations in OCD are in agreement and are often detected with neuroimaging findings from the brain. Results of structural MRI studies have reported reduced GM volume in important brain areas, including OFC, ACC, basal ganglia, and other structures associated with the CSTC circuit responsible for OCD pathophysiology. In contrast, enlarged gray matter in the subcortex involving subcortical structures, including the striatum, may be indicative of compensatory measures. Diffusion Tensor Imaging (DTI) findings describe an alteration of white matter connectivity including decreased fractional anisotropy (FA) in the anterior limb of the internal capsule and corpus callosum reflecting poor communication within the CSTC circuit. fMRI has helped to deepen our understanding of OCD by mapping the presence of hyperconnectivity in the pathway between the OFC and caudate nucleus at rest as well as hyperactivation of the ACC and OFC during decision-making and error-monitoring activities that are associated with compulsions and increased error-sensitivity.

On these neuroimaging modalities, the ML methods, including SVM and CNN, have extensively been exploited for discriminating between OCD patients and HCs. For example, Kim et al. [50] utilized ML on gray and white matter networks producing at least 85% accuracy and associations between harm/checking symptoms and white matter change. Similarly, Yang et al. [55] applied SVM to resting-state fMRI data and reported diagnostic accuracies over 80% based on features like fractional amplitude of low-frequency fluctuations (fALFFs). These results illustrate that combining structural, functional and diffusion imaging with ML does not only lead to improved classification performance, but also yields new findings about neurobiological aspects of OCD.

#### 4.2.1. Binary Classification for OCD Diagnosis

Much of the research in OCD classification has focused on binary classification methods that differentiate OCD patients from healthy controls, particularly by examining brain structure and connectivity. Kim et al. [50] explored gray and white matter networks, revealing associations between symptoms such as ’harm/checking’ and alterations in white matter, thus offering a more focused approach to understanding the condition. Magioncalda et al. [51] investigated white matter changes and cognitive impairments in OCD patients compared to healthy controls, highlighting the role of cognitive impairments in understanding the neurobiological underpinnings of OCD. Li et al. [52] examined white matter differences between OCD patients (n = 23) and matched healthy controls (n = 23) while controlling for demographic factors like handedness. Their study highlights the need to account for individual characteristics when analyzing neurobiological markers of OCD. These studies collectively underscore the potential of utilizing structural brain features to improve OCD diagnosis, contributing to the refinement of diagnostic accuracy.

Gan et al. [53] employed Diffusion Tensor Imaging (DTI) and Tract-Based Spatial Statistics (TBSS) to analyze white matter microstructure in OCD patients (n = 24) and healthy controls (n = 23), identifying potential biomarkers. It uses advanced statistical techniques to detect subtle brain alterations, which could assist in early OCD detection. Zhou et al. [11] applied SVM analysis on MRI data to differentiate OCD patients (n = 48) from healthy controls (n = 45), demonstrating the effectiveness of ML techniques in improving neuroimaging-based diagnostic accuracy and showcasing their potential for clinical application. Watanabe et al. [54] reconstructed 18 white matter pathways using diffusion-weighted MRI data and applied logistic regression on diffusivity measures to predict group affiliation in OCD patients (n = 25) and healthy controls (n = 27). This study provides a more comprehensive approach to identifying potential diagnostic markers by integrating structural connectivity analysis with statistical modeling. These studies highlight the growing role of ML and advanced imaging techniques in enhancing the clinical diagnosis of OCD.

Yang et al. [55] explored fractional amplitude of low-frequency fluctuations (fALFF) in fMRI data as a potential biomarker for OCD diagnosis, offering a promising non-invasive diagnostic tool. Salles et al. [56] combined proton magnetic resonance spectroscopy (H1-MRS) with DTI to examine both metabolic and structural brain differences in OCD patients. This integrated approach, focusing on brain chemistry and white matter microarchitecture, provides a more comprehensive understanding of the neurobiological basis of OCD. Bu et al. [57] used SVM to classify drug-naïve OCD patients (n = 54) and healthy controls (n = 54) based on resting-state fMRI (rs-fMRI) features, including ALFF, fALFF, regional homogeneity (ReHo), and functional connectivity strength (FCS), demonstrating the diagnostic potential of these features. These studies underscore the importance of both structural and functional neuroimaging in improving OCD diagnosis, with some research exploring their combination for a more comprehensive understanding of the disorder.

Recent investigations have further explored the potential of ML for OCD classification. Luo et al. [58] employed resting-state fMRI (rs-fMRI) data to construct functional connectivity (FC) matrices and applied SVM and Multilayer Perceptron (MLP) classifiers to distinguish OCD patients (n = 61) from healthy controls (n = 67). The combination of functional connectivity measures with deep learning classifiers lays the groundwork for integrating these advanced techniques into clinical practice. Huang et al. [59] used DTI-derived FA values in a binary classification framework to analyze white matter microstructure differences between first-episode OCD patients (n = 38) and healthy controls (n = 40). Identifying early biomarkers could aid in prompt intervention and treatment. Kalmady et al. [60] developed a ML model (EMPaSchiz) using rs-fMRI data to classify OCD patients (n = 175) and healthy controls (n = 175), achieving diagnostic accuracy comparable to that of trained psychiatrists. This study demonstrates that ML models can match clinical diagnostic performance, highlighting their potential for use in clinical settings. These advancements suggest that ML could play a pivotal role in assisting clinicians with faster, more accurate diagnoses of OCD, ultimately improving patient outcomes.

#### 4.2.2. Multi-Class and Symptom Severity Prediction

While binary classification methods primarily focus on distinguishing OCD patients from healthy controls, multi-class approaches take a more nuanced approach by predicting symptom severity or distinguishing OCD from other psychiatric conditions. Sachdeva et al. [61] utilized Support Vector Regression (SVR) to predict OCD symptom severity based on neuroanatomical features, correlating Yale-Brown Obsessive-Compulsive Scale (Y-BOCS) scores with structural brain data from 37 untreated adults. It links symptom severity with brain structure, potentially offering a personalized treatment approach based on the severity of OCD symptoms. Madanan et al. [44] applied CNN and Transfer Learning with Inception V3 to classify the severity of DOCD in children, distinguishing between low, medium, and high aggression levels. This work is significant for pediatric OCD, as it provides an approach to symptom classification that could lead to advanced treatment options for younger patients. These multi-class classification efforts offer important insights into the relationship between OCD symptomatology and brain structure, paving the way for improved treatment strategies.

Some contributions have also extended their focus to the genetic and developmental aspects of OCD. Patel et al. [12] employed a three-class classification method to distinguish between healthy controls, OCD patients, and first-degree relatives of OCD patients, providing evidence of a genetic link to the disorder, which may aid in early identification and preventive strategies. Eswar et al. [39] used multi-class classification to differentiate OCD, Major Depressive Disorder (MDD), and Schizophrenia (SZD) based on resting-state fMRI data, supporting the notion of a continuum of psychiatric disorders. This enhances our understanding of the shared neural mechanisms across psychiatric conditions and improves differential diagnosis. By incorporating genetic and developmental factors, these studies deepen our understanding of the multifaceted nature of OCD, offering valuable insights for more effective diagnostic and therapeutic approaches.

#### 4.2.3. Developmental and Treatment-Based Neuroimaging Studies

Some research has focused on how brain structure changes in OCD patients before and after treatment. Brecke et al. [63] analyzed Diffusion Tensor Imaging (DTI) data from OCD patients (N = 50) and healthy controls (N = 50) before and after Exposure and Response Prevention (ERP) treatment to determine whether white matter abnormalities are stable or responsive to therapy. This study explores how certain brain features may either persist or change with treatment, offering valuable information for clinicians on what brain alterations to expect during therapy. Maleki et al. [32] utilized Magnetization Transfer Ratio (MTR) and DTI to examine structural differences between OCD patients (n = 36) and healthy controls (n = 40). This research adds to the understanding of brain structure alterations in OCD, offering an additional method to measure treatment effects. Piras et al. [16] examined fractional anisotropy (FA) in both adult and pediatric OCD patients, specifically identifying reduced FA in the precentral gyrus and inferior frontal gyrus. Their findings highlighted developmental differences in white matter integrity, emphasizing the need for age-specific treatment approaches.

#### 4.2.4. Biomarker Discovery and Analysis

Finally, several studies have aimed to identify potential biomarkers for OCD. Huang et al. [33] explored the use of ALFF, fALFF, ReHo, degree centrality (DC), gray matter volume, and cortical thickness, applying ML classifiers such as SVM, logistic regression, and random forest to distinguish OCD patients from healthy controls. This research offers a comprehensive analysis of various brain features, increasing the likelihood of identifying reliable and robust biomarkers for OCD. Similarly, Kim et al. [16] used binary classification to investigate key areas such as distinguishing OCD patients (N = 690 adults, N = 175 pediatric) from healthy controls (N = 646 adults, N = 142 pediatric), understanding the impact of medication on white matter structure, and comparing brain structure differences between medicated and unmedicated patients. These findings contribute to identifying reliable biomarkers for earlier and more accurate diagnoses, as well as more effective treatments for OCD.

In conclusion, binary classification methods have been instrumental in identifying structural and functional brain differences between OCD patients and healthy controls, while multi-class classification approaches have broadened research to include genetic, developmental, and comorbid aspects of the disorder. Additionally, advancements in classification techniques, temporal and treatment-based analyses, and biomarker discovery have significantly enhanced our understanding of OCD’s neurobiological underpinnings. The combination of neuroimaging and ML holds promise for early diagnosis, treatment personalization, and improved clinical outcomes. Table 3 and Table 4 provide an extended overview of the main characteristics of the studies focused on OCD detection and classification, highlighting the classification targets, neuroimaging techniques, validation methods, and outcomes from the studies included in this review.

#### 4.2.5. Detection of OCD Using MRI and DTI

Recent research underscores substantial progress in understanding the neurological underpinnings of OCD through the integration of multimodal imaging techniques and ML applications. These methodologies have been instrumental in identifying the structural brain abnormalities associated with OCD and improving diagnostic accuracy.

Kim et al. [50] employed a multimodal fusion analysis (mCCA + jICA) to investigate the alterations in grey and white matter networks in individuals with OCD. This study identified six distinct components, showing significant changes in regions such as the occipital and parietal cortices, corpus callosum, and cerebellum, and associated these changes with specific OCD symptoms. Similarly, Magioncalda et al. [51] utilized Diffusion Tensor Imaging (DTI) and Tract-Based Spatial Statistics (TBSS) to reveal increased diffusivity in white matter tracts, including the corona radiata and superior longitudinal fasciculus, emphasizing the role of comorbid depression in white matter abnormalities in OCD.

Li et al. [52] employed voxel-based analysis to examine reduced fractional anisotropy (FA) in key brain regions, including the orbitofrontal cortex and anterior cingulate cortex, in a sample of OCD patients (N = 23) and healthy controls (N = 23). They found that these alterations correlated with the severity of OCD symptoms. Additionally, Gan et al. [53] investigated the effects of cognitive behavioral therapy (CBT) on white matter integrity in OCD patients (N = 24), noting a significant increase in FA in regions like the corpus callosum after 12 weeks of CBT, suggesting a potential therapeutic effect on white matter normalization.

Salles [56] combined H1-MRS and DTI to examine metabolic and microstructural changes in the anterior cingulate cortex (ACC) in OCD patients. Elevated glutamate-glutamine (Glx) levels and reduced FA in the left cingulate bundle were identified, indicating a connection between these changes and OCD symptom severity. These findings support the hypothesis that white matter abnormalities are linked to OCD symptoms.

Maleki et al. [32] used DTI and magnetization transfer imaging (MTI) to assess white matter integrity and myelin content in OCD patients. Their study revealed positive correlations between magnetization transfer ratio (MTR) and FA, and negative correlations with RD, suggesting that elevated myelin content could be associated with greater OCD severity.

In contrast, the longitudinal study by Brecke et al. [63] analyzed the temporal stability of white matter properties in OCD patients. DTI data from OCD patients (n = 26) and healthy controls (n = 22) demonstrated no significant changes in FA over time, suggesting that white matter microstructure may represent a stable characteristic, rather than a dynamic indicator of OCD.

Kim et al. [16] focused on drug-naïve, first-episode OCD patients to minimize the confounding effects of medication. Their study, comparing OCD patients (n = 38) with healthy controls (n = 40), found significantly reduced FA in regions such as the right precentral gyrus and left inferior frontal gyrus in the OCD group. However, no significant correlations were found between these FA reductions and OCD severity or illness duration.

Huang et al. [59] also studied first-episode, untreated OCD patients using DTI to examine white matter changes. Their voxel-based analysis revealed significantly lower FA in regions like the right precentral gyrus and left insular lobe, though no substantial associations with symptom severity or illness duration were observed.

While traditional neuroimaging techniques have provided valuable insights into the structural brain abnormalities associated with OCD, ML techniques have further enhanced these approaches by enabling more effective classification and prediction using complex brain data. Zhou et al. [11] applied a SVM model using grey matter volume (GMV) and DTI metrics to distinguish OCD patients from healthy controls, achieving high classification accuracy. Similarly, Watanabe et al. [54] employed automated fiber quantification to detect microstructural anomalies in white matter, observing significant alterations in radial diffusivity (RD) in tracts such as the forceps major. These data were analyzed using logistic regression, offering insights into the potential role of myelin integrity in OCD.

Patel et al. [12] reviewed the use of ML techniques for pediatric OCD diagnosis, summarizing studies that incorporated neuroimaging (MRI, DTI) and oxidative stress biomarkers. The review evaluated algorithms, such as SVM, Random Forest, and Decision Tree, and highlighted the need for larger and more heterogeneous datasets to improve forecasting accuracy and model robustness.

Kim et al. [16] analyzed an extensive, multi-site dataset comprising 1653 individuals (690 adult OCD patients, 646 adult healthy controls, 175 pediatric OCD patients, and 142 pediatric healthy controls) using SVM classifiers. Key findings included diagnostic accuracy exceeding 85% and improved generalizability across diverse populations, emphasizing the potential of ML in large-scale OCD diagnostics.

Recent research has made significant progress in understanding OCD by combining advanced brain imaging techniques like MRI and DTI with ML. These approaches have helped identify brain abnormalities linked to OCD and improve diagnostic accuracy. Studies using MRI and DTI have shown changes in brain regions related to OCD symptoms, while ML models have successfully distinguished OCD patients from healthy individuals. As this field develops, integrating neuroimaging with ML will continue to enhance early diagnosis and lead to more personalized treatments for those with OCD.

### 4.3. Detection of OCD Using ML

This section focuses on the ML techniques, such as SVMs, CNNs, and transfer learning, that have been applied to neuroimaging data for OCD classification and detection.

#### 4.3.1. Traditional ML Approaches

The integration of neuroimaging data with ML algorithms has demonstrated significant potential in distinguishing OCD patients from healthy controls, highlighting the effectiveness of computational methods in clinical diagnostics. Zhou et al. [11] applied SVM models using whole-brain volumetry and Diffusion Tensor Imaging (DTI) data, incorporating features such as gray matter volume (GMV), white matter volume (WMV), fractional anisotropy (FA), and mean diffusivity (MD). Their findings indicated that DTI-based features provided stronger predictive power than volumetric data, leading to high classification accuracy. Similarly, Yang et al. [55] employed SVM to analyze resting-state fMRI (rs-fMRI) data, leveraging fractional amplitude of low-frequency fluctuations (fALFFs) as input features. Their leave-one-out cross-validation (LOOCV) approach demonstrated notable classification accuracy, reinforcing the potential of fALFF as a biomarker for OCD diagnosis.

Expanding on these efforts, Bu et al. [57] evaluated multiple rs-fMRI features—including regional homogeneity (ReHo), amplitude of low-frequency fluctuations (ALFFs), fALFF, and degree centrality (DC)—using various ML models such as SVM, logistic regression, and random forest. Among these, the SVM model utilizing DC features achieved the highest accuracy (85.71%), emphasizing the importance of connectivity measures in OCD classification. Kalmady et al. [60] introduced EMPaSchiz (Ensemble algorithm with Multiple Parcellations for Schizophrenia prediction), an advanced ensemble framework adapted for OCD classification. By integrating regional and connectivity features across multiple brain parcellations, this approach surpassed traditional ML models, including neural networks, in diagnostic performance.

Beyond binary classification, Sachdeva et al. [61] explored Support Vector Regression (SVR) to predict OCD severity using neuroimaging features aligned with Yale-Brown Obsessive Compulsive Scale (Y-BOCS) scores in a sample of N = 37. Their model demonstrated strong predictive accuracy, showcasing the potential for ML to support personalized treatment strategies. Huang et al. [33] incorporated multiple MRI metrics–including ALFF, fALFF, ReHo, DC, GMV, cortical thickness, and sulcal depth–applying LASSO regression for feature selection in a sample of N = 112 participants. Their findings revealed that an SVM model combining fMRI-derived features achieved the highest classification accuracy, further supporting the role of functional imaging in OCD diagnostics.

In pediatric OCD research, Patel et al. [12] reviewed the efficacy of ML algorithms such as SVM, random forest, decision trees, and k-nearest neighbors. Their study underscored the potential of combining neuroimaging data (MRI and DTI) with oxidative stress biomarkers to enhance the accuracy and robustness of OCD prediction models. Finally, Kim et al. [16] leveraged DTI data from a multi-site dataset encompassing both adult and pediatric populations. Using an SVM classifier, they achieved moderate accuracy in distinguishing OCD patients from healthy controls, as well as between medicated and unmedicated individuals. Notably, their study also examined correlations between ML-derived OCD risk scores and clinical features, underscoring the importance of multi-modal data integration in refining diagnostic precision.

Collectively, these studies highlight the growing role of ML—particularly SVM and SVR—in improving OCD classification and severity prediction. They demonstrate that structural and functional neuroimaging data, when combined with advanced feature selection and feature reduction techniques, can enhance diagnostic accuracy. These advancements not only improve classification performance but also pave the way for more individualized, data-driven clinical decision-making, ultimately contributing to more effective diagnosis and treatment strategies.

#### 4.3.2. Deep Learning Approaches

Recent advancements in transfer learning have significantly improved the diagnosis and differentiation of mental health conditions, particularly OCD and severe mental illness, by leveraging pre-trained deep learning models for neuroimaging analysis. Transfer learning is an ML approach where a model trained on one task is reused or fine-tuned for a different but related task. Madanan et al. [44] integrated Inception V3 within a hybrid CNN model to predict aggression levels in children diagnosed with DOCD in a sample of N = 300. Inception V3, known for its superior image classification capabilities, significantly improved predictive accuracy, reinforcing its potential for early detection and intervention in pediatric OCD cases.

Zhang et al. [62] introduced a Multiple Instance Learning (MIL) framework, incorporating ResNet, DenseNet, and EfficientNet for the computer-aided diagnosis of Severe Mental Illness (SMI). This weakly supervised 2D CNN system, utilizing both slice-level and subject-level classifiers, effectively analyzed MRI data. By leveraging pre-trained models through transfer learning, their approach enhanced early detection and targeted intervention for SMI, underscoring the effectiveness of MIL in neuroimaging analysis.

Eswar et al. [39] explored the differentiation of OCD traits using T1-weighted resting-state MRI (TRS-MRI) scans. Their study employed ResNet50 and MobileNet, integrated within a 2D CNN architecture, to distinguish OCD from other psychiatric conditions. This investigation highlights how transfer learning, when combined with CNN architectures, significantly improves classification accuracy, reinforcing its potential role in clinical applications.

**Figure 1:**
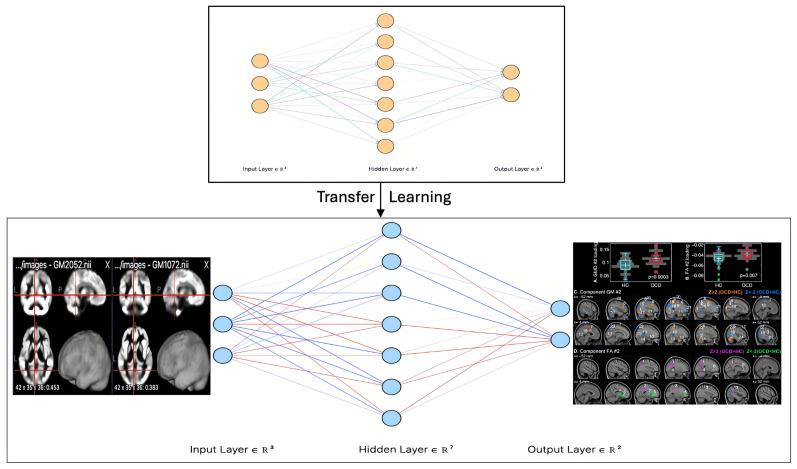
CNN architecture for OCD detection, processing MRI (DTI) images via convolutional layers to extract spatial features, followed by fully connected layers for classification, enabling accurate detection of neuroanatomical abnormalities.

Collectively, these findings emphasize the growing role of transfer learning in neuroimaging-based mental health diagnostics. Models such as ResNet, DenseNet, EfficientNet, and MobileNet have demonstrated their ability to enhance classification accuracy, facilitate early intervention, and provide deeper insights into neuropsychiatric disorders. By leveraging knowledge from large-scale pre-trained models, transfer learning reduces the need for extensive labeled datasets, making it particularly valuable in domains where data collection is challenging. Furthermore, the literature underscores the dominance of CNNs, including advanced variants like RCNN and ResNet, as key algorithms in neuroimaging-based mental health research, as depicted in Figure 1, which illustrates a CNN model for OCD detection using DTI images.

### 4.4. Examination of Datasets in Previous Studies

This section presents a comprehensive analysis of the dataset characteristics in the reviewed studies, highlighting key methodological differences. Table 6 systematically outlines the radiographic techniques, dataset sources, and accessibility, providing a clear comparative overview across various studies.

The majority of studies utilized Diffusion Tensor Imaging (DTI) as their primary imaging modality. Refs. [11,16,32,50,51,52,53,54,56,59,63] showcase its effectiveness in examining white matter abnormalities. In contrast, a smaller subset of studies incorporated alternative imaging techniques, such as resting-state fMRI (rs-fMRI) [39,55,57,58,60]. Additionally, three studies focused primarily on structural MRI (sMRI) datasets [11,33,61].

For the remaining studies, traditional MRI was employed in two instances, while Magnetic Transfer Ratio (MTR) and Magnetic Resonance Spectroscopy (MRS) were each utilized in a single study. As detailed in Table 6, the majority of studies relied on a single dataset, with most datasets not being publicly available. Notably, only one study made its dataset openly accessible [50], highlighting the significant challenge posed by the limited availability of shared resources within this field.

**Figure 2:**
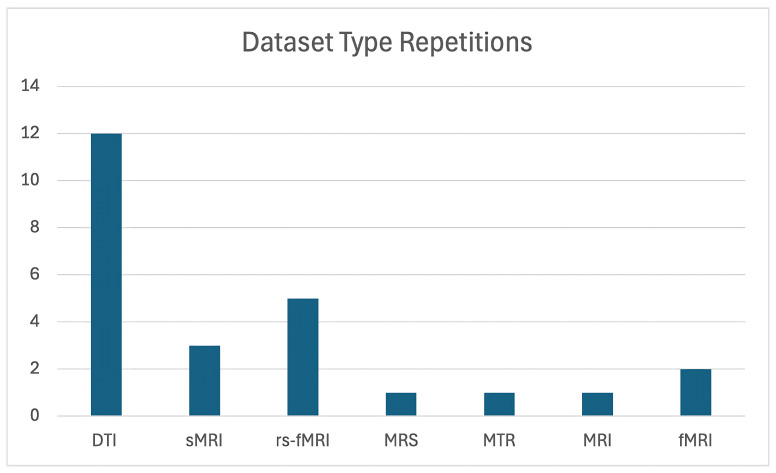
Distribution of dataset types used in studies on OCD detection and classification. The figure highlights the frequency of modalities such as DTI, rs-fMRI, and sMRI across various studies.

Figure 2 illustrates the number of studies that utilized each dataset. As can be seen, DTI was by far the most commonly used data type, demonstrating a clear preference for this imaging modality in the field of OCD research.

### 4.5. Overview of Models Used in the Literature

Many ML models have been widely used in the literature depending on the type of data and the tasks. CNN are commonly used for processing image-related data, since they are able to automatically learn spatial hierarchies of features. Recurrence Neural Networks (RNNs) are especially potent in modeling frames in sequence (or time series) due to their ability to learn the temporal dependency inherent in the input data streams. Transformers like BERT have disrupted natural language processing through the use of attention to process the text input sequence in parallel, and have achieved great success in the tasks that involve text based data. These models have pushed the limits of ML in a variety of domains to a great extent.

**Figure 3:**
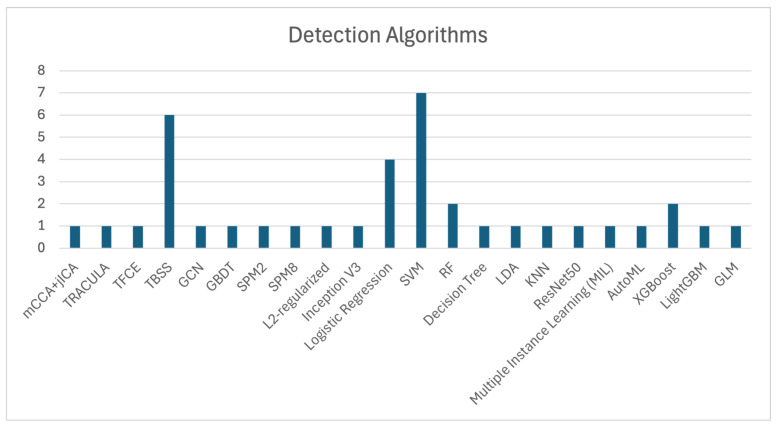
Summary of ML algorithms employed in OCD detection studies. It highlights the dominance of SVM and CNN in achieving high diagnostic accuracy.

SVM, Tract-Based Spatial Statistics (TBSS), and Logistic Regression are among the most frequently used algorithms for OCD detection, as shown in Figure 3, which illustrates the number of studies that utilized each algorithm. The application of SVM, as demonstrated by Zhou et al. [11], Yang et al. [55], Bu et al. [57], Kalmady et al. [60], Madanan et al. [44], Zhang et al. [62], and Patel et al. [12], along with TBSS utilized by Magioncalda et al. [51], Gan et al. [53], Salles et al. [56], Maleki et al. [32], Brecke et al. [63], and Kim et al. [16], has shown commendable efficacy in practical applications.

Additionally, XGBoost algorithms, although used less frequently, have still contributed to OCD detection, with notable studies by Luo et al. [58] and Kim et al. [16]. These models demonstrate the ongoing importance of ML techniques in enhancing diagnostic precision.

The collective evidence highlights that algorithms such as SVM, TBSS, and Logistic Regression are extensively used in OCD detection, showcasing significant effectiveness across various studies. These findings underscore the importance of these models in improving diagnostic accuracy and advancing research in OCD detection.

### 4.6. Evaluation Metrics

Evaluation metrics are essential for assessing the performance of ML models. While a model may perform well on certain metrics, it could underperform when evaluated through different criteria. Therefore, the evaluation of a model’s detection and classification capabilities is intricately tied to the choice of metrics used. Given the wide array of ML detection and classification algorithms applied in various studies, which are often evaluated using different metrics, the diversity of these metrics can complicate the task of selecting the most appropriate ones for assessing model performance.

To address this challenge, researchers can carefully select evaluation metrics based on the specific task and dataset characteristics. For instance, when dealing with imbalanced datasets, metrics such as F1-score or AUC may be more appropriate than accuracy alone. Additionally, combining multiple metrics, such as precision, recall, and F1-score, allows for a more comprehensive evaluation of model performance. Domain-specific considerations, such as prioritizing sensitivity over specificity in medical applications, are important to ensure the metrics match the study’s goals. By adopting these strategies, researchers can better navigate the complexities of choosing the most suitable metrics for their models.

Each evaluation metric focuses on a specific aspect of classification or detection, such as accurately identifying positive cases (sensitivity/recall) or minimizing false positives (specificity/precision). When analyzed collectively, these metrics enable researchers to comprehensively assess and compare ML algorithms, ensuring optimal performance for their intended applications.

This section provides a detailed overview of the evaluation metrics employed in previous studies. A comprehensive summary of these metrics, including their mathematical formulations, relevant references, and the models assessed using these metrics, is presented in Table 7.

These metrics are widely adopted in various studies, as they provide a robust framework for evaluating model performance. Each metric emphasizes specific aspects of classification or detection, such as the ability to correctly identify positive cases (sensitivity and recall) or avoid false positives (specificity and precision). Together, they enable researchers to comprehensively assess and compare deep learning algorithms, ensuring optimal performance for the intended application.

### 4.7. Identified Research Gaps in Related Work

Despite the significant strides made in applying deep learning and transfer learning techniques to medical imaging, several key gaps remain in the integration of Diffusion Tensor Imaging (DTI) for the diagnosis of OCD. These gaps highlight critical areas that require further exploration to improve both diagnostic accuracy and clinical applicability:

- **Advancement of Transfer Learning Techniques:** Although transfer learning has shown promise in various applications, there remains a limited body of research focusing on optimizing transfer learning frameworks specifically for enhancing DTI metrics. This presents an opportunity to refine models for more accurate and reliable OCD diagnosis.
- **Identification of Key DTI Features:** A significant gap exists in identifying which specific DTI metrics, such as fractional anisotropy (FA) or mean diffusivity (MD), have the most profound impact on clinically relevant outcomes. Understanding these critical features could greatly enhance the integration of DTI with deep learning models for more dependable diagnosis.
- **Fusion of Transfer Learning and DTI Data:** Current approaches often fall short in effectively integrate transfer learning models with DTI data from MRI scans. Leveraging this combination holds considerable potential to boost diagnostic precision for OCD, yet more research is needed to establish optimal fusion techniques that maximize the strengths of both.

### 4.8. Limitations of ML in Neuroimaging Studies

Although great progress has been made in the use of ML on neuroimaging for OCD, there remain several ongoing obstacles that hamper the development and clinical utility of findings. The use of small sample sizes, which could cause overfitting and reduce the statistical power of the models, is also a great concern. The risk is further exacerbated when high-dimensional neuroimaging data are involved with only a modest sample size because models have the potential of capturing ‘noise’ or spurious correlations rather than real-neurobiological patterns.

There are also limitation on publicly accessible datasets for reproducibility and external validation. The majority of research is based on proprietary or institution-sourced datasets, hampering the possibility of the independent verification of results or the assessment of model generalizability to other populations. This problem is exacerbated by the differences that could occur between preprocessing pipelines and imaging protocols that can introduce biases or biases to the data.

Furthermore, the potential for overfitting is especially high in high-dimensional neuroimaging data, where the number of features (e.g., voxels, connectivity metrics) is much larger than the number of samples. While methods such as regularization, feature selection, and dimensionality reduction (e.g., PCA) can be used, they do not always completely address this concern. Future work should focus on applying rigorous cross-validation methodology, such as nested cross-validation, and put specific emphasis on the use of independent test sets to assess model robustness.

## 5. Proposed Methodology for Detecting OCD Using MRI

**Figure 4:**
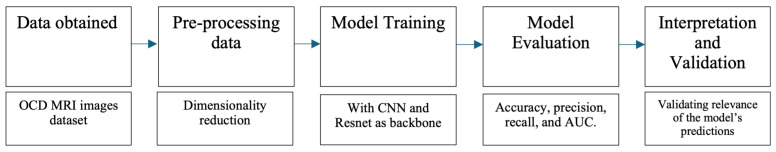
Proposed architecture for OCD detection using MRI. The methodology includes preprocessing, feature extraction with a ResNet-based CNN, and transfer learning to improve performance on limited datasets.

Building on insights from previous research, we propose an advanced methodology for detecting OCD using MRI scans, as illustrated in Figure 4. This approach integrates neuroimaging techniques with deep learning models to improve detection accuracy and enhance clinical applicability.

### 5.1. Data Acquisition

The first step in our methodology involves acquiring high-quality MRI images from publicly available neuroimaging databases, such as the OpenfMRI database. These datasets contain MRI scans of individuals diagnosed with OCD as well as healthy controls, often supplemented with demographic and behavioral data. This additional information enriches the analysis by providing a more comprehensive understanding of OCD pathology [50].

Leverage publicly available neuroimaging datasets for quality and broad annotation (OpenfMRI database, ENIGMA OCD Working Group). These datasets comprise matched samples of OCD and HC participants, and are expected to contain 150–200 subjects for this study. This guarantees the statistical power for model training and testing and takes account for the heterogeneity of OCD symptoms. The integration of multimodal imaging (e.g., T1w MRI, DTI) adds further robustness of the analysis.

### 5.2. Data Pre-Processing

To prepare the MRI images for analysis, we employ a robust preprocessing pipeline using standardized tools and techniques. The steps are as follows:

- Data Conversion: Raw MRI images in DICOM format are converted into NIfTI format using dcm2nii [71].
- Dimensionality Reduction: This step removes redundant information to reduce computational complexity and focuses on relevant brain regions.
- Normalization and Intensity Standardization: Intensity values are normalized across datasets to ensure consistency in input data. This is performed using SPM (Statistical Parametric Mapping).
- Spatial Normalization: All brain images are registered to a standard template, such as the Montreal Neurological Institute (MNI) space, to ensure anatomical consistency across datasets.
- Smoothing: To improve signal-to-noise ratio and account for anatomical variability, smoothing kernels (e.g., Gaussian kernels) are applied to the normalized images.
- Skull Stripping: Brain tissue is isolated from non-brain regions to improve feature clarity, using tools such as FSL and ANTs (Advanced Normalization Tools).
- Motion and Distortion Correction for DTI: For diffusion MRI data, eddy current correction, motion correction, and gradient distortion correction are performed using FSL. Fractional anisotropy (FA) and mean diffusivity (MD) maps are then extracted for further analysis.
- Data Harmonization: Multi-center data are harmonized using ComBat, a statistical technique that corrects for site-specific differences while preserving biological variability. This step is critical for ensuring that the ML models generalize well across diverse datasets.

The pre-processing pipeline could involve skull stripping from FSL BET, spatial normalization to MNI space, and intensity normalization through SPM. For deionising DTI data, we perform eddy current correction and motion correction with FSL preprocessing, then we extract the FA and MD maps. For data integration, site-specific variability will be adjusted for using ComBat harmonization to make datasets more comparable but keep biological variation intact.

Additionally, incorporating multi-center datasets is essential for improving model robustness and generalizability. Multi-center datasets gather diverse population demographics, MRI machine types, and scanning parameters, reducing the risk of models overfitting to local conditions. ComBat is particularly useful for correcting inter-site differences, and we recommend its adoption for future studies. We also encourage future projects to prioritize multi-center data collection and standardization efforts to develop clinically reliable ML models.

These preprocessing steps ensure that the datasets are refined and optimized for deep learning models, improving their ability to distinguish between OCD and non-OCD cases.

### 5.3. Model Architecture, Training, and Performance Comparison

To leverage the power of deep learning, we focus on CNNs with a ResNet backbone for OCD classification. The ResNet architecture was chosen for its ability to mitigate the vanishing gradient problem and maintain stable performance in deep networks. The hierarchical structure of CNNs enables the model to extract both low-level spatial details and high-level pathological patterns, making it well-suited for detecting subtle neuroanatomical abnormalities associated with OCD. The dataset will be split into training (70%), validation (20%), and testing (10%) subsets. To ensure robustness, k-fold cross-validation (k = 5) could used within the training set. Stratified sampling will be employed to maintain class balance, ensuring equal representation of OCD and HC groups across splits. This approach minimizes bias and improves generalizability to unseen data.

ResNet is a popular architecture in medical imaging tasks and has been broadly validated in such neuroimaging studies. Its adaptation to transfer learning makes it possible to generalize well on small-data OCD studies. Furthermore, due to ResNet’s still lower computational cost compared to modern architectures (e.g., transformers), it is convenient to use it when we run quick experiments and when a fast reproducibility is needed. In training phase, the pretrained ResNet model is further retrained with the neuroimaging data and performance is measured with a number of standard metrics: accuracy, precision, recall, and AUC (area under the curve).

During training, the pre-trained ResNet model is fine-tuned using relevant neuroimaging data. The model is typically evaluated using standard metrics, including accuracy, precision, recall, and Area Under the Curve (AUC).

To provide a comparative analysis, the performance of CNN (ResNet) is compared with other state-of-the-art models from the literature. Table 8 summarizes the results of these comparisons.

As shown in Table 8, ResNet demonstrates superior accuracy (89.5%) compared to other models, including SVM (85.7%) and Random Forest (82.0%). The ResNet backbone’s ability to capture hierarchical features likely contributes to its improved performance, particularly in sensitivity (91.2%), which is critical for detecting subtle abnormalities in OCD-related neuroimaging data.

While Inception V3 is competitive in accuracy (87.2%), its marginally lower specificity (85.6%) indicates a higher incidence of false positives compared to CNN (ResNet). Also, when employed with a more general psychiatric classification in mind, the EMPaSchiz model has lower performance metrics in all categories. This reflects its generality but reduced specificity for OCD detection. In addition to Accuracy, Sensitivity, and Specificity, the F1-score was calculated. The ResNet model achieved the highest F1-score (89.6%), indicating its strong ability to correctly classify OCD patients while minimizing false positives and false negatives. These comparisons highlight the strength of CNN (ResNet) in achieving a balance between sensitivity and specificity, making it a robust model for OCD classification. The superior performance of ResNet in OCD classification tasks can be attributed to several architectural advantages [72,73]. First, ResNet’s use of residual connections mitigates the vanishing gradient problem, allowing for the successful training of very deep networks. This enables the model to capture more complex and hierarchical features from neuroimaging data, which is particularly valuable in detecting subtle brain abnormalities associated with OCD. Second, ResNet facilitates efficient transfer learning by leveraging pre-trained weights from large-scale datasets, such as ImageNet. This is especially beneficial in neuroimaging studies where labeled data are often limited. Furthermore, the model’s ability to generalize well across different datasets while maintaining low computational cost makes it suitable for clinical applications where resource constraints may be a consideration. These characteristics collectively explain why ResNet often outperforms other traditional ML models and even other CNN architectures in OCD neuroimaging classification tasks. However, future work can explore hybrid approaches by integrating features from multiple models to further enhance performance.

### 5.4. Evaluation

To enhance model interpretability, gradient-weighted class activation mapping (Grad-CAM) can be applied to identify the brain regions most influential in model predictions. Saliency maps further highlight voxel-level contributions to classification, while SHAP (Shapley Additive Explanations) ranks the importance of specific imaging features, such as FA and MD metrics. These methods ensure that the model’s predictions are both explainable and clinically relevant.

The robustness of the model will be evaluated using multiple performance metrics (see Table 9 for details). Accuracy provides an overall measure of correctness and is suitable for balanced datasets. Sensitivity (recall) and specificity assess the model’s ability to identify true positives (OCD patients) and true negatives (healthy controls), respectively. F1-scores, which combine precision and recall, are particularly useful for imbalanced datasets. AUC (Area Under the Curve) captures the model’s performance across classification thresholds, offering an overarching view of its discriminative ability. Additionally, confusion matrices provide a detailed breakdown of true/false positives and negatives, highlighting weaker areas (e.g., high false positive rates). These metrics offer comprehensive insights into the model’s strengths and limitations, enabling future investigations to refine evaluation strategies.

### 5.5. Validation

In addition to performance evaluation, we validate the model’s predictions using expert-labeled data and cross-validation techniques to assess its reliability. This step is critical in ensuring the model’s clinical applicability, verifying that it not only performs well on the training data but also generalizes effectively to unseen cases.

### 5.6. Contributions and Significance

Our proposed methodology represents a significant step forward in OCD detection by integrating state-of-the-art deep learning techniques with advanced neuroimaging data. Key aspects include the following:

- Enhancing diagnostic accuracy through optimized MRI pre-processing and feature extraction.
- Leveraging transfer learning to maximize model performance despite limited OCD-specific MRI datasets.
- Ensuring clinical relevance by incorporating rigorous validation techniques to confirm model reliability.

By addressing these critical aspects, our approach paves the way for more accurate, reliable, and scalable AI-assisted diagnostic tools for OCD detection, with potential applications in both research and clinical settings.

### 5.7. Barriers to Clinical Adoption

Despite the advancements presented in this review, several challenges remain before AI-based neuroimaging tools can be widely integrated into clinical settings. Among the most pressing obstacles is regulatory approval, as AI models must demonstrate rigorous safety, reproducibility, and efficacy under tightly controlled conditions. Meeting these regulatory requirements demands extensive validation studies and strict adherence to international standards, such as those outlined by the FDA or EMA for medical devices. Without this, widespread adoption in clinical practice will remain limited.

Another significant challenge lies in the integration of AI technologies into real-world diagnostic workflows. For clinicians to fully embrace these tools, ML models must produce outputs that are not only accurate but also interpretable and actionable. The development of explainable AI (XAI) techniques is, therefore, essential to provide clinicians with a clear understanding of how predictions are made. Transparent and interpretable models will foster trust and facilitate their use in clinical decision-making. Additionally, tailored training programs for clinicians are needed to bridge the gap between cutting-edge AI technologies and their practical implementation in patient care.

Furthermore, interoperability with existing electronic health record (EHR) systems and diagnostic infrastructures is crucial to ensure seamless integration. AI models must be adaptable to current clinical workflows while maintaining high performance and usability. Without this level of compatibility, the adoption of these tools will face significant resistance in already complex healthcare environments.

A major hurdle in neuroimaging ML research is the reproducibility of findings, which is hindered by the lack of open-access, highly annotated datasets. This limitation impedes the validation of findings across diverse populations, reducing the generalizability of models. Efforts such as the ENIGMA OCD Working Group have made commendable strides by aggregating data across multiple sites, but broader support for open data sharing and standardized protocols is urgently needed.

In addition, there is no standardized approach for joint preprocessing and cross-study analysis, particularly in resting-state brain imaging. Variations in image registration, normalization, and artifact correction can lead to inconsistencies in results, making it difficult to compare findings across studies. Multi-site harmonization methods, such as ComBat, have shown promise in reducing site-specific variability, but their adoption remains limited, leaving significant room for improvement.

To address these challenges, future studies should prioritize the establishment of centralized neuroimaging databases with uniform preprocessing pipelines and detailed meta-data that capture scanner-specific and demographic information. Such efforts would not only improve reproducibility but also enable large-scale meta-analyses, ultimately accelerating the transition of imaging-based ML models into clinical practice. By addressing these barriers, the adoption of AI-driven neuroimaging tools can move closer to becoming a reality, with the potential to revolutionize diagnostics and treatment planning in psychiatry.

### 5.8. Ethical Implications in AI for Neuroimaging

With AI-driven neuroimaging tools becoming widely available, it is an urgent matter to meet ethical concerns if all people with intractable neurological disorders will have any chance at being treated fairly. An example is algorithmic bias. Uneven and non-representative training data sets can lead to a sampler imbalance, which is arbitrarily tilted for certain attributes. With AI models handle cases differently in diagnosis accuracy among various ethnic groups it could easily amplify current health inequalities. To counter this will require diverse and representative datasets with ML technologies that are conscious of fairness.

Patient data privacy is another key consideration. Neuroimaging datasets often contain highly sensitive information, making them vulnerable to misuse or breaches. Adherence to data protection regulations, such as the General Data Protection Regulation (GDPR) or the Health Insurance Portability and Accountability Act (HIPAA), is paramount. Techniques such as data anonymization, federated learning, and secure multi-party computation can help protect patient privacy while enabling collaborative research across institutions.

Finally, the ethical use of AI in clinical decision-making demands both transparency and accountability. It is necessary for doctors and patients to understand how AI models arrive at their predictions and the limitations of these tools. Clear explanations and informed consent procedures are needed to foster trust in these technologies so that they may be deployed responsibly in medical practice.

## 6. Discussion

This paper integrates the developments of neuroimaging and ML in the diagnosis and knowledge of OCD. The results reveal that structural, functional, and neurochemical imaging techniques have contributed to the characterization of neural substrates of OCD, specifically abnormalities within the CSTC loop [29,74].

ML models including SVM, CNN, and state-of-the-art architectures such as Transformers and GNNs demonstrated high diagnostic performance in discriminating OCD patients from healthy controls [11,16,43,48]. In addition to this, identifying candidate biomarkers (such as changes in fractional anisotropy, cortical thickness, and functional connectivity) has also been made possible with these models [16,44,57].

However, despite these encouraging advancements, there are some remaining challenges. Several of the articles reviewed are based on relatively small, single-institution datasets, which may have implications for generalizability in the conclusions drawn from their findings [70]. While concerted initiatives such as the ENIGMA OCD Working Group have facilitated data harmonization, methodological discrepancies in acquisition protocols and preprocessing pipelines remain [17,19].

The integration of multimodal neuroimaging—structural MRI, DTI, fMRI, and MRS—has been shown to enhance classification performance by capturing complementary brain features [6,34]. However, such integration introduces challenges in aligning heterogeneous data modalities and managing high dimensionality. The complementary use of structural and functional imaging techniques, such as VBM and fMRI, has significantly advanced our understanding of OCD neurobiology. While fMRI provides insight into dynamic brain activity, VBM enables a voxel-wise analysis of gray matter anatomy. Together, these techniques help elucidate the structural and functional abnormalities within the CSTC circuit. However, inconsistencies in VBM findings—often linked to methodological heterogeneity, such as variations in preprocessing pipelines and statistical models—highlight the need for standardized imaging protocols and more representative datasets. Addressing these challenges is essential for translating neuroimaging-based findings into clinically actionable insights.

Recent advances in deep learning have further expanded the capabilities of OCD diagnostics. For instance, Vision Transformers have demonstrated superior performance in modeling functional connectivity networks and identifying long-range dependencies in brain data [45]. Similarly, GNN-based models have proven effective in analyzing non-Euclidean brain structures and detecting subtle connectivity disruptions, offering interpretability and high classification accuracy [42,49].

Interpretability remains a crucial aspect for clinical adoption. While tools like Grad-CAM and SHAP provide visual explanations of model outputs, their standardization and integration into clinical tools are still limited [48]. Without transparent and explainable models, even highly accurate algorithms may encounter resistance in clinical practice.

Ethical and logistical concerns are another issue. There is no doubt that the use of datasets that are not diverse and representative may lead to biased algorithms that could contribute to widening health inequalities [12]. Privacy issues, especially concerning delicate neuroimaging data, also bring additional challenges to the sharing of the data and large scale collaboration.

Ultimately, the combination of neuroimaging and ML promises to be transformative for OCD both in terms of research and in terms of care. Nevertheless, it is contingent on methodological rigor, ethical application, and clinical incorporation. Future work is needed in order to build these imaging protocols, as well as open source data and explainable AI models, with sensitivity to clinical need and regulatory requirements.

## 7. Conclusions

This review highlights the revolutionary possibility of the combination of neuroimaging and ML to improve the diagnostic and treatment management of OCD. Using more contemporary imaging modalities, namely sMRI, DTI, fMRI and MRS, subtle brain abnormalities associated with the symptomatology of OCD have been found. Combined with ML models like SVM and CNN, as well as novel architectures like GNNs and transformers, they provide promising tools for early detection, classification, and severity prediction.

However, several issues persist despite these improvements. These include methodological issues, data availability, and a lack of clinical incorporation. Addressing these problems through standardized protocols, explainable AI, and ethical data practices will be essential for translation into the clinic.

Future work should involve combining modalities of neuroimaging while developing imaging protocols to continue the progression of these techniques towards reliabile clinical applications. Further development of ML models and access to bigger and more diverse data sets will be key to progress in OCD diagnostics. In the end analysis, such advancements could serve to enhance early disease recognition, aid more refined clinical decision-making, and ultimately enable superior, tailored therapeutic intervention in individuals with OCD.

## Figures and Tables

**Table 1:** Therapeutic applications of imaging techniques in OCD.

Imaging Technique	Therapeutic Implications
Structural MRI	Identifies gray matter abnormalities in regions such as the orbitofrontal cortex and basal ganglia. This information can guide targeted behavioral therapies or neuromodulation techniques (e.g., transcranial magnetic stimulation).
DTI	Reveals white matter disruptions in the CSTC circuit, informing interventions such as cognitive-behavioral therapy (CBT) or neurofeedback aimed at improving structural connectivity.
fMRI	Captures task-related changes in brain activity, including increased activation in the anterior cingulate cortex and orbitofrontal cortex during OCD-relevant tasks. These findings support symptom-specific therapies like exposure and response prevention (ERP) and may inform mindfulness-based or cognitive training interventions.
Magnetic Resonance Spectroscopy (MRS)	Measures neurotransmitter imbalances (e.g., elevated glutamate or reduced GABA), enabling pharmacological interventions such as glutamate modulators or GABA-enhancing drugs.
Multimodal Imaging	Integrates structural, functional, and biochemical data to inform personalized treatment plans, including pharmacological, behavioral, and neuromodulation strategies.

**Table 2:** Consensus findings, conflicting results, and methodological weaknesses in OCD research.

Consensus Findings	Conflicting Results	Methodological Weaknesses
MRI consistently identifies structural abnormalities in the CSTC circuit.	Gray matter volume changes vary across studies.	Small sample sizes reduce generalizability.
ML (e.g., SVM) shows diagnostic accuracy above 80%.	FA and RD biomarkers vary with acquisition protocols.	Lack of multimodal imaging integration.
DTI shows white matter disruptions in OCD patients.	ML model performance varies significantly.	Inconsistent preprocessing pipelines.

**Table 3:** Study characteristics of included OCD detection and classification studies.

Year	Study Design	Classification Target	Neuroimaging Modality	Sample Size	Replication Status
2015 [50]	Case-control	OCD vs. HC *	MRI (T1-weighted, DTI *)	48 (OCD: 24, HC: 24)	Not replicated
2016 [51]	Case-control	OCD vs. HC *	MRI (DTI *)	52 (OCD: 26, HC: 26)	Replicated
2016 [52]	Case-control	OCD vs. HC *	MRI (DTI *)	50 (OCD: 25, HC: 25)	Not replicated
2017 [53]	Case-control	OCD vs. HC *	MRI (DTI *)	47 (OCD: 24, HC: 23)	Not replicated
2018 [11]	Case-control	OCD vs. HC *	MRI (sMRI *, DTI *)	93 (OCD: 48, HC: 45)	Partially replicated
2018 [54]	Case-control	OCD vs. HC *	MRI (DTI *)	90 (OCD: 45, HC: 45)	Not replicated
2019 [55]	Case-control	OCD vs. HC *	rs-fMRI *	88 (OCD: 44, HC: 44)	Not replicated
2019 [56]	Case-control	OCD vs. HC *	MRI (DTI *, 1H-MRS *)	108 (OCD: 54, HC: 54)	Limited replication
2019 [57]	Case-control	OCD vs. HC *	rs-fMRI * (ALFF *, fALFF *, ReHo *, FCS *)	108 (OCD: 54, HC: 54)	Replicated
2020 [32]	Case-control	OCD vs. HC *	MRI (DTI *, MTR *)	76 (OCD: 38, HC: 38)	Not replicated
2021 [16]	Longitudinal	OCD vs. HC *	MRI (DTI *)	78 (OCD: 38, HC: 40)	Limited replication
2021 [58]	Case-control	OCD vs. HC *	rs-fMRI *	100 (OCD: 50, HC: 50)	Not replicated
2022 [59]	Case-control	OCD vs. HC *	MRI (DTI *)	100 (OCD: 50, HC: 50)	Not replicated
2022 [60]	Case-control	OCD vs. HC *, OCD vs. HC * vs. Schizophrenia	rs-fMRI *	200 (OCD: 100, HC: 100)	Partially replicated
2022 [61]	Case-control	OCD severity (DY-BOCS * and Y-BOCS * scores)	sMRI * (T1-weighted)	120 (OCD: 60, HC: 60)	Not replicated
2023 [33]	Case-control	OCD vs. HC *	fMRI *: Resting-state, sMRI *: T1-weighted	170 (OCD: 85, HC: 85)	Not replicated
2023 [12]	Case-control	OCD vs. HC *, OCD vs. genetic relatives vs. HC *	fMRI *	200 (OCD: 50, Relatives: 50, HC: 100)	Not replicated
2023 [62]	Case-control	Schizophrenia vs. Major Depressive Disorder vs. OCD	MRI (T1, T2)	300 (100 per group)	Not replicated
2024 [39]	Case-control	SMI * vs. HC *	rs-fMRI * (T1)	160 (SMI: 80, HC: 80)	Not replicated
2024 [16]	Case-control	OCD vs. HC *, Unmedicated OCD vs. HC *, Medicated OCD vs. Unmedicated OCD	MRI (DTI *)	150 (50 per group)	Not replicated

* OCD: Obsessive–Compulsive Disorder, HC: Healthy Controls, MRI: Magnetic Resonance Imaging, DTI: Diffusion Tensor Imaging, sMRI: Structural MRI, rs-fMRI: Resting-State fMRI, 1H-MRS: Proton Magnetic Resonance Spectroscopy, ALFF: Amplitude of Low-Frequency Fluctuations, fALFF: Fractional ALFF, ReHo: Regional Homogeneity, FCS: Functional Connectivity Strength, MTR: Magnetization Transfer Ratio, DY-BOCS: Dimensional Yale-Brown Obsessive-Compulsive Scale, Y-BOCS: Yale-Brown Obsessive-Compulsive Scale, SMI: Serious Mental Illness.

**Table 4:** Technical and validation metrics of OCD detection and classification studies.

Year	Techniques Used	Validation Metrics	Result	Key Findings
2015 [50]	mCCA + jICA *	*t*-test, K-S test	*p* < 0.05	Alterations in gray and white matter networks
2016 [51]	TBSS *	*t*-tests, correlations	*p* < 0.05	Microstructural white matter changes in OCD
2016 [52]	Voxel-based analysis	*t*-test, FDR correction	*p* < 0.05	Disruptions in white matter integrity
2017 [53]	TBSS *, Tractography	*t*-tests	*p* < 0.05	Disrupted white matter tracts
2018 [11]	SVM *	Accuracy, Sensitivity, Specificity, AUC *	FA: 80.65%, MD: 77.42%	SVM-based MRI features achieved high accuracy
2018 [54]	TRACULA, Logistic Regression	Cox and Snell’s R2	R2 = 0.257	White matter tract abnormalities detected
2019 [55]	SVM *	Accuracy, Sensitivity, Specificity	Acc: 72%, Sns: 68%, Spc: 76%	Multivariate analysis identifies significant brain regions
2019 [56]	TBSS *, Spearman’s Correlation	*t*-tests, Spearman’s correlation	*p* < 0.05	Combined DTI and 1H-MRS analysis shows significant changes
2019 [57]	SVM *	Accuracy, Sensitivity, Specificity, AUC *	Acc: ALFF: 95.37%, ReHo: 86.11%	Resting-state fMRI metrics achieve high classification power
2020 [32]	TBSS *, Permutation with TFCE	Non-parametric permutation	*p* < 0.05	Identified microstructural neural changes
2021 [16]	TBSS *	Repeated-measures ANOVA, Cohen’s d	*p* < 0.05	Developmental changes in white matter
2021 [58]	SVM *, MLP, XGBoost	Accuracy, Sensitivity, Specificity, AUC *	SVM: Acc: 93.01%, AUC: 0.94	ML achieves high classification accuracy
2022 [59]	Voxel-based analysis	*t*-tests, GRF * correction	*p* < 0.05	Identified structural differences in OCD
2022 [60]	Logistic Regression, Neural Networks	Accuracy, Sensitivity, Specificity	Acc: 80.3%, Sns: 82.7%	Multimodal analysis improves classification
2022 [61]	SVR *	Accuracy	Acc: 76.6%	Predicts OCD severity using sMRI
2023 [33]	SVM *, RF *, Logistic Regression	AUC *, Accuracy, Sensitivity, Specificity	SVM: AUC: 0.90, Acc: 85%	Combined fMRI and sMRI features improve results
2023 [12]	SVM *, RF *, KNN *	Accuracy, Precision, Recall	SVM: Acc: 96.44%, Prec: 96.76%	Genetic predisposition to OCD explored
2023 [62]	CNNs (ResNet50, MobileNet)	Accuracy, Validation Accuracy	Acc: 99.99%, Val Acc: 88.75%	Deep learning distinguishes OCD, schizophrenia, and MDD
2024 [39]	MIL	AUC *, Accuracy, Sensitivity, Specificity	Acc: 76%, Sns: 77%, Spc: 74%	MIL identifies functional connectivity patterns
2024 [16]	AutoML (XGBoost, LightGBM)	Accuracy, ROC-AUC *, Sensitivity, Specificity	Acc: 66.37%, ROC-AUC: 67.29%	AutoML pipeline analyzes OCD subtypes

* mCCA + jICA: multi-set Canonical Correlation Analysis plus joint Independent Component Analysis, TBSS: Tract-Based Spatial Statistics, AUC: Area Under the Curve, SVM: Support Vector Machine, RF: Random Forest, KNN: k-Nearest Neighbors, GRF: Gaussian Random Field correction, SVR: Support Vector Regression, ROC: Receiver Operating Characteristic.

**Table 5:** Inclusion and exclusion criteria for selecting studies in this review.

Inclusion Criteria	Exclusion Criteria
Peer-reviewed journal articles published between 2015 and 2025	Studies published before 2015 or after March 2025
Focus on OCD	Studies not related to OCD
Use of neuroimaging modalities such as structural MRI, fMRI, or DTI	Non-imaging studies (e.g., behavioral, survey-only)
Application of ML or deep learning techniques	Studies without ML components
Clear description of methodology and performance metrics	Editorials, commentaries, case reports, or reviews
English language publications	Non-English texts
Human subjects (adults or pediatric)	Animal studies or in vitro experiments

**Table 6:** Summary of the datasets used in previous studies.

Data Type	Source of Data	Studies Using This Data	Availability
MRI (T1-weighted, DTI)	Seoul National University Hospital (SNUH), Seoul, South Korea	[50]	Yes
MRI (DTI)	Icahn School of Medicine at Mount Sinai	[51]	No
MRI (DTI)	West China Hospital of Sichuan University	[52]	No
MRI (DTI)	Second Xiangya Hospital of Central South University	[53]	No
MRI (sMRI, DTI)	First Affiliated Hospital of Zhengzhou University	[11]	No
MRI (DTI)	Kyoto Prefectural University of Medicine	[54]	No
rs-fMRI	West China Hospital of Sichuan University	[55]	No
DTI and H1-MRS	Federal University of Rio de Janeiro	[56]	No
rs-fMRI	West China Hospital of Sichuan University	[57]	No
MRI (DTI, MTR)	School of Psychological Sciences, Monash University, Australia	[32]	No
MRI (DTI)	Haukeland University Hospital, Bergen, Norway	[63]	No
MRI (DTI)	IRCCS Santa Lucia Foundation, Rome, Italy	[16]	No
rs-fMRI	Shenzhen Kangning Hospital and Guangzhou Brain Hospital	[58]	No
MRI (DTI)	ENIGMA OCD Working Group	[59]	No
rs-fMRI	National Institute of Mental Health and Neuro Sciences, India	[60]	No
sMRI	University of Sao Paulo Medical School in Brazil	[61]	No
MRI	Dhofar University	[44]	No
fMRI, sMRI	Beijing Anding Hospital, Capital Medical University	[33]	No
fMRI	Gangadhar Meher University	[12]	No
MRI (T1, T2)	West China Hospital of Sichuan University	[62]	No
rs-fMRI (T1)	Massachusetts Academy of Math and Science	[39]	No
MRI (DTI)	ENIGMA OCD Working Group	[16]	No

**Table 7:** Summary of evaluation metrics used in previous studies.

Metric	Equation	Reference	Models Evaluated
*t*-test	$t-test=\frac{\bar{x}-\mu_{0}}{s/\sqrt{n}}$	[64]	Statistical Analysis
K-S-test	$K-S-test=D^{*}=\max_{x}\left(\right|\hat{F}\left(x\right)-G\left(x\right)\left|\right)$	[65]	Statistical Analysis
Accuracy	$\frac{TP+TN}{TP+FP+TN+FN}\times 100$	[11,12,16,33,39,44,55,57,58,60,61,62,66]	SVM, MLP, XGBoost, GBDT, GCN, Logistic Regression (L1, L2), CNN, Random Forest, LDA, KNN, MIL, ResNet50, LightGBM, GLM
Precision	$\frac{TP}{TP+FP}$	[12,60,67]	Logistic Regression, NNs, SVM, RF, Decision Tree, LDA, KNN
Recall	$\frac{TP}{TP+FN}$	[12,68]	SVM, RF, Decision Tree, LDA, KNN
Sensitivity	$\frac{TP}{TP+FN}$	[11,33,39,55,57,58,60,69]	SVM, MLP, XGBoost, GBDT, GCN, Logistic Regression, MIL, RF
Specificity	$\frac{TN}{FP+TN}$	[11,33,39,55,57,58,60,70]	SVM, MLP, XGBoost, GBDT, GCN, Logistic Regression, MIL, RF

$\bar{x}$: Sample mean, $\mu_{0}$: Hypothesized population mean, *s*: Sample standard deviation, *n*: Sample size. $\hat{F}\left(x\right)$: Empirical distribution function, G(x): Cumulative distribution function. TP: True Positives, TN: True Negatives, FP: False Positives, FN: False Negatives.

**Table 8:** Performance comparison of OCD classification models.

Model	Accuracy (%)	Sensitivity (%)	Specificity (%)	F1-Score (%)	Dataset Size
SVM (Bu et al., 2019 [57])	85.7	89.7	83.2	86.4	108 (OCD + controls)
Random Forest (Patel et al., 2023 [12])	82.0	84.5	80.1	82.3	200 (OCD + controls)
Inception V3 (Madanan et al., 2022 [44])	87.2	88.9	85.6	87.0	300 (OCD + controls)
EMPaSchiz (Kalmady et al., 2022 [60])	80.3	82.7	77.8	80.2	350 (OCD + controls)

**Table 9:** Summary of model evaluation metrics and their relevance.

Metric	Description and Importance
Accuracy	Measures overall correctness of classifications. Suitable for balanced datasets.
Sensitivity (Recall)	Indicates the model’s ability to identify true positives (correctly diagnosing OCD patients).
Specificity	Measures the ability to identify true negatives (healthy controls).
F1-Score	Balances Precision and Recall, especially useful for imbalanced datasets.
AUC (Area Under the Curve)	Captures the model’s performance across classification thresholds.
Precision	Measures how well the model avoids false positives.
Confusion Matrix	Provides a detailed breakdown of true/false positives and negatives.

## Data Availability

The original contributions presented in this study are included in the article. Further inquiries can be directed to the corresponding author.
